# Adjectives improve color perception in visually impaired people through multisensory stimulation

**DOI:** 10.3389/fpsyg.2026.1718682

**Published:** 2026-05-18

**Authors:** Ching-Yi Wang, Yi-Jung Chuo

**Affiliations:** Department of Creative Product Design, Asia University, Taichung, Taiwan

**Keywords:** auditory, color adjective, multisensory, olfactory, tactile, visually impaired people

## Abstract

Visually impaired individuals, particularly those who are totally blind, cannot rely on vision to perceive colors and instead depend on other sensory modalities. Color adjectives may serve as semantic representations that support their understanding of the surrounding world. This study investigates how visually impaired individuals construct color experiences and meanings through multisensory stimulation. In this study, semi-structured interviews were conducted with six participants to elicit descriptions of nine colors (red, orange, yellow, green, blue, purple, white, gray, and black) based on tactile, olfactory, auditory, and emotional cues. A follow-up questionnaire study involving fifteen participants was then conducted to examine the recognition and applicability of the identified color adjectives. The results indicate that participants showed preferences for black, green, yellow, and red. Multisensory stimulation effectively elicited diverse color-related adjectives, which could be categorized into five types: emotional, taste, appearance, time, and abstract. Auditory stimuli were particularly effective in eliciting emotional adjectives. Overall, the findings suggest that visually impaired individuals' understanding of color can be constructed through cross-sensory associations, particularly via tactile and auditory experiences.

## 1 Background

Color adjectives refer to descriptive words that characterize colors through sensory, emotional, or conceptual associations rather than solely through visual attributes. Prior studies suggest that such adjectives play a key role in the formation of color concepts, especially for individuals with limited or absent visual experience, as color knowledge in these populations is largely acquired through language, embodied experience, and multisensory stimulation (Eardley and Pring, 2014; Saysani et al., 2018; van Paridon et al., 2021). From a broader perspective, color perception has been shown to systematically influence human psychological functioning, including affective responses, motivation, and cognition, even when color information is conveyed symbolically or linguistically rather than visually (Elliot and Maier, 2014). Current research on visually impaired people has many applications of color adjectives, including daily life, education, art and design. In daily life, such as shopping, choosing food, and identifying maps, the application of color adjectives helps visually impaired people adapt to life more conveniently. In education, the application of color adjectives helps visually impaired people better understand colors, images, etc. In art, the application of color adjectives helps visually impaired people express and experience colors more effectively (Begeske et al., 2023). In design, color adjectives can be applied to create designs that are more accessible to visually impaired people (van Paridon et al., 2021). However, the vocabulary development of the visually impaired people is limited by traditional learning methods and environments (Galiano and Portalier, 2011; Zhang et al., 2019), resulting in a smaller vocabulary than sighted people of the same age possess. Previous reviews have emphasized that reduced access to visually grounded lexical input constrains the richness and precision of descriptive language in individuals with visual impairment, highlighting the central role of linguistic mediation in color concept acquisition (Galiano and Portalier, 2011). The vocabulary development of visually impaired people requires specific learning strategies and teaching methods, and sensory stimulation such as tactile, olfactory, hearing, and texture are used to help them identify and distinguish colors (Lewis et al., 2013; Marian et al., 2021; Ramsamy-Iranah et al., 2016; Zhang et al., 2019). This situation may be explained by the fact that visually impaired people develop color-related understanding primarily through non-visual sensory processing, as auditory and tactile inputs often play a more prominent role in their perceptual experiences than visual information (Elbert et al., 1995; Zhao et al., 2018). Therefore, an important research direction is using the different senses of the visually impaired people to stimulate the perception and vocabulary description of color.

Color adjectives can provide more detailed visual information, helping the visually impaired better perceive the world (Eardley and Pring, 2014; Saysani et al., 2018; Marian et al., 2021). Previous research has shown that color perception relies on both sensory input and higher-level cognitive processing, with attributes such as luminance and contrast playing a central role in object and scene recognition (Gegenfurtner and Rieger, 2000). Surprisingly, despite their absence of visual exposure to color, congenitally blind individuals often exhibit consistent color associations, which they acquire through language-based learning (van Paridon et al., 2021). Color–emotion associations have been widely documented in both perceptual and symbolic contexts. For example, red has been consistently linked to danger, warning, and urgency across behavioral and cognitive studies, even when presented without direct visual cues (Elliot and Maier, 2014; Pravossoudovitch et al., 2014). Beyond single-color effects, environmental studies have demonstrated that light and color can systematically influence psychological mood and affective states across different cultural contexts (Küller et al., 2006). Moreover, cross-cultural and linguistic evidence suggests that color lexicons are shaped jointly by environmental exposure and cultural conventions, allowing stable color semantics to emerge even in the absence of direct sensory experience (Josserand et al., 2021). In particular, blind and sighted people have more common color understandings for natural categories (e.g., bananas) and artifacts with functional colors (e.g., stop signs) than for artifacts with non-functional colors (e.g., cars) (Kim et al., 2021). Many previous studies have found that the visually impaired could obtain color information through multisensory stimulation. Believe that the implementation of tactile color pictograms has the potential to enhance the appreciation of artwork for individuals with visual impairments. In addition, some studies discovered evidence suggesting that the relationship between music and color might stem from an emotional connection (Barbiere et al., 2007; Sun et al., 2018). Normally, cheerful songs tended to be associated with brighter colors like yellow, red, green, and blue, while sad songs were often linked with gray hues (Jonas et al., 2017). These findings have certain implications for visually impaired people to perceive and describe colors through auditory means. Similar studies, such as Saysani et al. (2018), explore how visually impaired people describe emotions through color. They asked the visually impaired people to smell different smells and describe the associated colors and emotions. The results showed that the color adjectives and emotional words mentioned by visually impaired people were similar to those of sighted people, but also that the visually impaired people used more negative emotional words. This result may be because the visually impaired have a strong negative reaction to threatening sounds (such as dog barking, car horn, etc.) (Zhao et al., 2018), such as those suggesting depressed, angry (angry), restless (uneasy), anxious (anxious), nervous (nervous), and restless (restless), as well as uncertainty about the surrounding environment, thereby causing strong negative feelings. Eardley and Pring (2014) explored the correlation between color adjectives produced by those visually impaired and visually intact subjects under different sensory stimulations. The results show that both visually impaired and sighted people can use their auditory and tactile senses to generate color adjectives and discover correlations between the two. Marian et al. (2021) explored the influence of vision, hearing and tactility on color perception. Visually impaired people were asked to identify colors and shapes while listening to sounds or touching objects. The results show that auditory and tactile stimulation can affect visual perception and that the visually impaired are more sensitive in this regard than sighted people. With similar results, a study by Sanchez et al. (2020) aimed to assess the emotional responses of visually impaired and sighted people to experienced musicians playing music. The findings suggest that visually impaired people's emotional responses to music may be related to their intense auditory experience and greater sensitivity to sound. These findings suggest that different sensory stimuli can still produce similar expressions of color adjectives in the human brain even in the absence of visual information. Such mappings are consistent with theories of crossmodal correspondences, which propose systematic associations between sensory dimensions (e.g., sound, touch, emotion) and color representations (Spence, 2011). In addition, the method of inducing adjectives for the visually impaired can include using items that are often touched (such as fruits). Lewis et al. (2013) used fruits (such as apples, bananas, cherries, grapes, lemons, oranges, papayas, pineapples, plums, strawberries, watermelons, etc.) as stimuli to build a color adjective library to help complete color descriptions of the color of objects. The above-mentioned studies emphasize the ability of the visually impaired to perceive and describe colors, as well as the differences in color vocabulary produced by different senses. Even in the case of visual impairment, other senses such as hearing and smell can still help the visually impaired to generate adjectives for colors. However, the vocabulary produced by different senses differs, and the meaning of some emotional words may vary from person to person. This study posits that there should be a more comprehensive investigation method for evoking color adjectives.

This study aims to explore general color understanding in visually impaired individuals from a pedagogical and developmental perspective. Using multisensory stimulation (tactile, olfactory, and auditory), the study elicits participants' descriptions of color experiences and emotional responses to clarify the underlying processes of color understanding and associated cognitive representations. Furthermore, the study integrates vocabulary generated across different sensory conditions to develop a structured framework that informs educational practices for individuals with visual impairments.

## 2 Methods

This study consists of two experiments. The first experiment adopts an exploratory qualitative approach to examine how visually impaired people's describe color-related experiences through non-visual sensory cues. The second experiment employs a questionnaire-based approach to investigate the recognition and applicability of the color adjectives extracted from the first experiment among participants with basic color knowledge. Accordingly, the two experimental components serve distinct research purposes and are not intended for direct quantitative comparison.

### 2.1 Experiment 1: description of color experience in visually impaired people

#### 2.1.1 Participants

This study conducted in-depth interviews with six visually impaired participants (two males; four females; mean age = 14.17 years; SD = 1.94 years) to explore their descriptions of color-related experiences and emotional responses.

According to the WHO classification of visual impairment, participants included individuals with Level 4 (blindness) and Level 5 (total blindness), with all cases being congenital in onset. Some participants retained minimal or residual light perception, while others had no light perception, as detailed in Appendix A. All participants had intact physical function and normal tactile ability.

Importantly, all participants were screened to ensure typical language development and the ability to comprehend and verbally express descriptive content; individuals with known language development delays were not included. Written informed consent was obtained from all participants prior to participation.

#### 2.1.2 Stimulus

The multisensory stimuli used in this study were designed based on crossmodal correspondence theory (Spence, 2011), which suggests systematic associations between sensory modalities and perceptual concepts such as color. Nine representative colors (red, orange, yellow, green, blue, purple, white, gray, and black) were selected, and each color was paired with corresponding tactile, auditory, and olfactory stimuli derived from commonly recognized sensory–semantic associations. To ensure experimental rigor and reproducibility, all stimuli were carefully standardized in terms of physical properties and presentation conditions (Table 1).

**Table 1:** Multisensory stimulus design and parameter standardization.

No.	Color	Tactile stimulus	Olfactory stimulus	Auditory stimulus
1	Red	Apple (Fuji; ~5–6 cm diameter; unpeeled; ~80% ripeness)	Apple scent (natural; ~1 cm; 3–5 s)	Fire truck siren (~60 dB; 5–10 s; mid–high frequency)
2	Orange	Orange (Navel orange; ~6–7 cm diameter; unpeeled; ripe)	Orange scent (~1 cm; 3–5 s)	Horn sound (~60 dB; 5–10 s; mid frequency)
3	Yellow	Banana (Cavendish; ~12–15 cm length; unpeeled; ripe)	Banana scent (~1 cm; 3–5 s)	Duck sound (~60 dB; 5–10 s)
4	Green	Leaves (Ficus microcarpa; ~5–6 cm length; fresh)	Leaf scent (~1 cm; 3–5 s)	Rustling leaves (~60 dB; 5–10 s; broadband)
5	Blue	Blueberry (*Vaccinium* spp.; ~1–2 cm diameter; fresh)	Blueberry scent (~1 cm; 3–5 s)	Sea wave sound (~60 dB; low–mid frequency)
6	Purple	Grape (Kyoho; cluster; ~2–3 cm diameter per fruit; unpeeled; ripe)	Grape scent (~1 cm; 3–5 s)	Soft music (~60 dB; neutral tone; 5–10 s)
7	White	Marshmallow (~3–4 cm diameter; soft texture)	Marshmallow scent (~1 cm; 3–5 s)	Rooster crowing (~60 dB)
8	Gray	Stone (~5–6 cm diameter; natural surface; moderate hardness)	Natural odor (~1 cm; 3–5 s)	Elephant sound (~60 dB; low frequency)
9	Black	Charcoal (~5–6 cm diameter; rough texture)	Natural odor (~1 cm; 3–5 s)	Nightingale sound (~60 dB; soft frequency)

For tactile stimuli, commonly available and standardized materials were selected. Fruit stimuli were specified by variety (e.g., Fuji apple, Cavendish banana, Kyoho grape), size (e.g., diameter or length), ripeness (~80% ripe), and preparation method (unpeeled), to maintain consistent tactile and olfactory characteristics. Non-food materials (e.g., stone and charcoal) were also controlled for size (~5–6 cm), surface texture, and natural properties.

Olfactory stimuli were standardized in terms of presentation distance (~1 cm) and duration (3–5 s). For natural materials, inherent scents were used without artificial enhancement to avoid introducing confounding olfactory effects.

Auditory stimuli were presented at approximately 60 dB, which is considered a comfortable listening level for children and unlikely to induce stress or aversive emotional responses. Given that individuals with visual impairments often exhibit heightened auditory sensitivity, controlling sound intensity was essential to avoid overstimulation and unintended emotional bias. All sounds were presented with a duration of 5–10 s and controlled frequency characteristics.

#### 2.1.3 Interview

The interview protocol followed a semi-structured format using open-ended questions. Sensory stimuli were provided solely to facilitate recall and verbal expression, while researchers avoided leading questions to minimize potential prompting bias.

The interview outline (Appendix B) was divided into four sections: (1) color basic concepts, (2) color sensory description, and (3) color emotional description. Among these, in terms of color sensory description, it is divided into (a) tactile, (b) olfactory, and (c) auditory, supplemented by stimuli (such as fruits) based on previous research recommendations (Kim et al., 2021; Lewis et al., 2013) to give participants stimulation and guidance, and arouse more experiences and emotional feelings linked to the colors.

#### 2.1.4 Procedure

**Figure 1:**
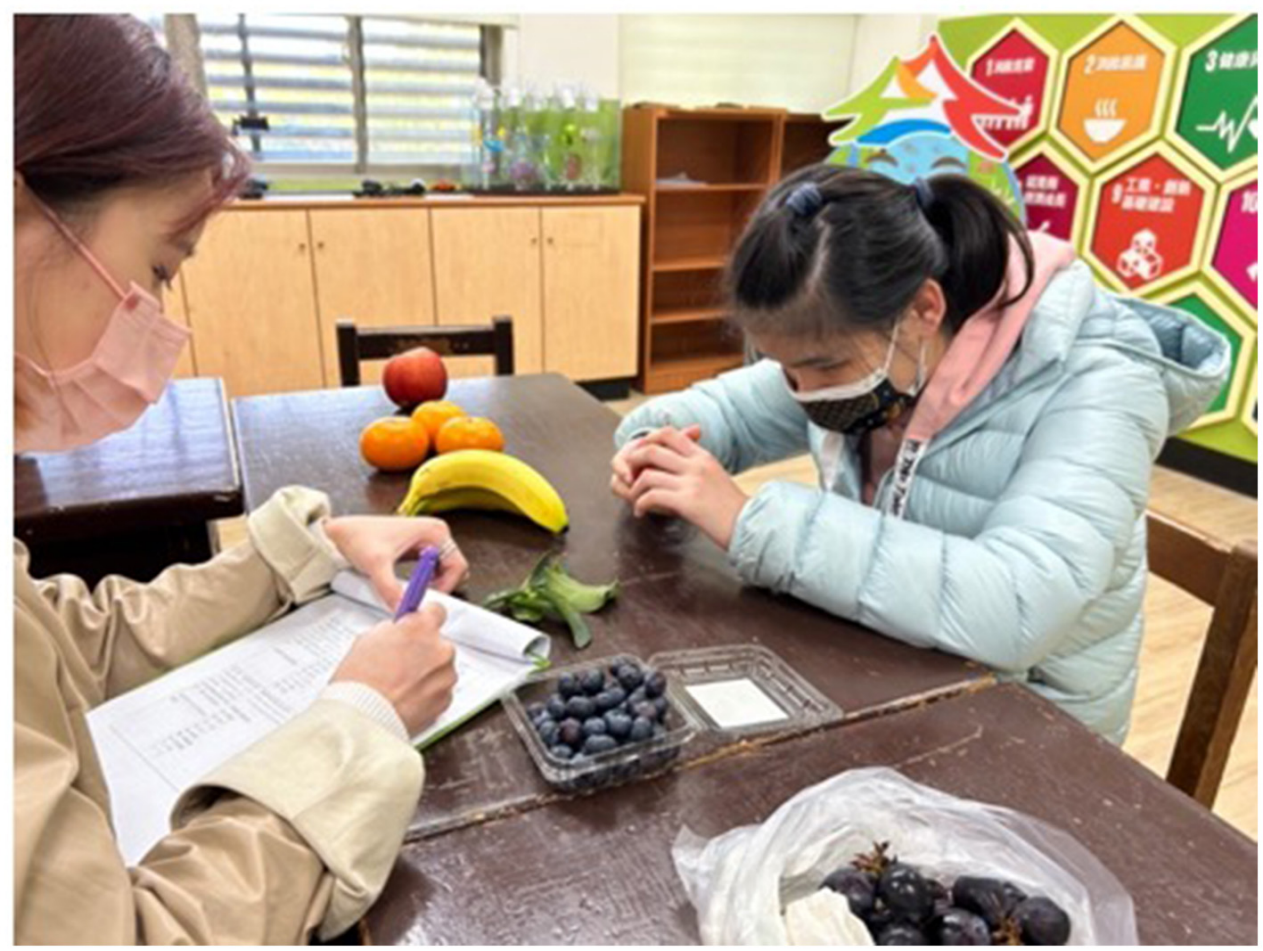
Actual experimental situation: visually impaired people touches stimulus.

Participant stayed in a quiet classroom to perform the experiment. The researchers asked them to sit in front of a table with stimuli. These stimuli (such as fruits, natural objects or music) were provided to participants by the researcher, and they were asked to use a designated sense (tactile, olfactory, auditory, and emotional descriptions; Figure 1) in reaction to the stimuli, and to try to recall and describe previous experiences. All of the participant's oral responses and expressions were photographed and recorded. Finally, the interview content was typed into a verbatim draft for subsequent data analysis.

#### 2.1.5 Data analysis

The interview data were analyzed using an inductive qualitative coding procedure focusing on color-related adjectives. The coding process consisted of three sequential step.

(1) Verbatim transcription and identification of analytic units: interview responses elicited under multisensory stimulation were transcribed verbatim. Each segment containing a complete and meaningful description was treated as an analytic unit.
(2) Extraction of salient adjectives: from each analytic unit, adjectives related to color experience were identified and extracted. When multiple adjectives appeared within a single statement, each adjective was coded independently (Appendix C). For example, under the tactile condition, the second participant described a stimulus as “It tastes sweet… I feel happy” (S2) During the coding process, the adjectives “sweet” and “happy” were both extracted as primary keywords. Adjectives with similar meanings were grouped into the same category based on semantic similarity and usage context across participants.
(3) Adjective categorization and frequency aggregation: the extracted adjectives were classified into five major categories: emotions (positive and negative), taste, appearance, time, and abstract (Table 2), and the frequency of each adjective across different sensory conditions was calculated and summarized. For example, “sweet” and “happy” were classified into the taste and positive emotion categories, respectively.

**Table 2:** Five types of color adjectives.

Type		Adjective
Emotion	Positive	“Confident,” “happy,” “beautiful,” “passionate,” “playful,” “kind,” “quiet,” “cute,” “peaceful,” “comfortable,” “gentle,” and “free”
	Negative	“Sad,” “scared,” “bloody,” “painful,” “noisy,” “dangerous,” “worried,” “melancholy,” “anxious,” “cold,” “warning,” “energetic,” and “gloomy”
Taste		“Sweet,” “sour and sweet,” “sour,” “juicy,” “bitter,” “salty,” “fragrant,” “smelly,” and “alcoholic”
Appearance		“Fresh,” “smooth,” “moist,” “plump,” “mature,” “rough,” “dark,” “invisible,” “dirty,” “hard,” “soft,” “light,” “heavy,” “huge,” and “crisp”
Time		“Seasonal” and “dawn”
Abstract		“Safe,” “natural,” “free,” “rural,” “family,” “outdoor,” “relaxed,” “calm,” “bright,” “peaceful,” “dreamy,” “distant,” “healthy,” “professional,” “handsome,” “low-profile,” “rare,” and “emergency”

### 2.2 Experiment 2: versatility of color adjectives

#### 2.2.1 Participants

Fifteen participants were asked to rate recognition of color adjectives (seven males; eight females; mean = 15.07 years; SD = 3.59 years). Participants have basic knowledge and experience of color. Detailed information on each participant's level of visual impairment is provided in Table B in Appendix A.

#### 2.2.2 Questionnaire

Participant assessed their recognition of the color adjectives selected in Experiment 1 (Table 3). Consider that they cannot easily distinguish between levels of degree. A 3-point response scale (1 = don't know/none; 2 = a little; 3 = know) was adopted to reduce cognitive load and facilitate reliable verbal responses in participant. Previous studies have shown that simplified rating scales improve comprehension and response quality in younger people and in populations with increased cognitive or sensory demands, particularly when abstract judgments are required (Coombes et al., 2021; Ruzek et al., 2020). During the experiment, the participants were asked to verbally address each adjective, which was then checked by the researcher.

**Table 3:** Color adjectives.

Color	Adjective
Red	Sweet, confident, bloody, emergency, fragrant, scared, crisp, painful, happy, energetic, passionate, sad
Orange	Sour and sweet, smooth, dangerous, juicy, noisy, seasonal, energetic, bitter, fragrant, healthy, sweet, happy, energetic, distant, sour, passionate, kind
Yellow	Fragrant, bright, safe, mature, playful, energetic, sad, sweet, happy, beautiful, worried, warning
Green	Natural, outdoor, safe, free, peaceful, beautiful, fragrant, rough, comfortable, happy, fresh, smooth, moist
Blue	Happy, outdoor, playful, relaxed, handsome, professional, sweet, soft, relaxed, smooth, beautiful, light, sour, and sweet
Purple	Sweet, seasonal, fragrant, scared, family, sad, alcoholic, gloomy, rare, sour and sweet, melancholy
White	Soft, dawn, cute, huge, noisy, fragrant, sweet, kind, rural, happy, gentle, dreamy, plump
Gray	Hard, gloomy, natural, happy, calm, heavy, sad, smelly, crisp, rough, smooth, kind, dirty
Black	Dark, quiet, low-profile, cold, invisible, sad, smelly, rough, anxious, hard, warning, dirty

#### 2.2.3 Data analysis

One-sample *t*-tests were conducted to compare the mean rating of each adjective for each color against a test value of 2. A significantly positive T value indicates that the adjective was rated significantly higher than the threshold, suggesting that participants tended to select the adjective as appropriate for the color. Conversely, a significantly negative T value indicates that the adjective was rated significantly lower than the threshold, suggesting that the adjective was generally not selected as appropriate.

## 3 Results

### 3.1 Sensory description of color

The extracted adjectives were organized into five categories, as shown in Table 2: emotion (positive and negative), taste, appearance, time, and abstract. Emotion-related adjectives reflected affective responses, taste and appearance captured sensory associations, time represented temporal references, and abstract included contextual or symbolic meanings. This categorization provides a structured framework for summarizing and comparing adjective use across multisensory stimulation.

Table 4 summarizes the distribution of color adjective categories elicited across different colors and multisensory stimulation, based on frequency counts (Table A in Appendix C) of adjectives produced by participants. Overall, the total number of elicited adjectives varied across colors, with orange (sum = 40) and red (sum = 32) generating the highest number of descriptors, followed by green (sum = 30) and white (sum = 29). In contrast, purple (sum = 17) elicited relatively fewer adjectives.

**Table 4:** Summary of frequency of color adjective categories across colors aggregated from multisensory conditions.

Conditions	Tactile							Olfactory							Auditory							Emotion							Sum
Color	P	N	T	A	T	B	Tot.	P	N	T	A	T	B	Tot.	P	N	T	A	T	B	Tot.	P	N	T	A	T	B	Tot.	
Red	2	0	2	1	0	0	**5**	1	0	7	2	0	0	**10**	0	6	0	0	0	0	**6**	5	2	0	0	0	4	**11**	**32**
Orange	1	0	7	1	1	0	**10**	0	0	4	0	0	1	**5**	0	3	0	0	0	3	**6**	9	0	0	0	0	10	**19**	**40**
Yellow	0	0	3	2	0	0	**5**	0	4	2	2	0	0	**10**	2	0	0	0	0	0	**2**	1	6	0	0	0	3	**10**	**25**
Green	0	0	0	5	0	0	**5**	0	0	1	2	0	2	**5**	0	0	0	2	0	3	**5**	9	0	0	1	0	5	**15**	**30**
Blue	0	0	0	4	0	0	**4**	0	0	4	0	0	0	**4**	4	0	0	0	0	3	**7**	2	0	0	0	0	5	**7**	**22**
Purple	0	0	2	0	1	0	**3**	0	0	8	0	0	0	**8**	0	2	0	0	0	0	**2**	0	2	0	0	0	2	**4**	**17**
White	0	0	1	6	0	0	**7**	1	0	5	0	0	0	**6**	0	2	0	0	2	2	**6**	6	0	0	0	0	4	**10**	**29**
Gray	0	0	0	11	0	0	**11**	0	0	4	1	0	1	**6**	0	0	0	1	0	0	**1**	2	2	0	0	0	2	**6**	**24**
Black	0	0	0	5	0	0	**5**	0	0	3	2	0	0	**5**	1	1	0	0	0	0	**2**	0	6	0	2	0	2	**10**	**22**
**Sum**							**55**							**59**							**36**							**92**	**242**

P, positive emotion; N, negative emotion; T, taste; A, appearance; T, time; B, abstract.

Appearance-related adjectives (type of A in Table 4) constituted the largest proportion, particularly under tactile conditions. This pattern was especially evident for gray, which showed a high concentration of appearance descriptors in the tactile modality, suggesting that surface texture, brightness, or material qualities played a central role in how this color was conceptualized.

Taste-related adjectives (type of T in Table 4) were predominantly associated with warm colors, particularly orange and Red, and were mainly elicited through tactile and olfactory stimulation. For example, orange showed a notably high frequency of taste descriptors (tactile: sum = 7; olfactory: sum = 4), reflecting strong associations between color and food-related sensory experiences.

In contrast, emotional adjectives (types of P and N in Table 4) were more frequently elicited under auditory and emotion-based conditions than under tactile or olfactory stimulation. Notably, red and black elicited both positive and negative emotional descriptors, indicating emotionally polarized interpretations of these colors. Yellow, however, showed relatively few negative emotional descriptors, aligning with its generally positive affective connotation within the sample.

Abstract adjectives (Type of B in Table 4) appeared less frequently overall and were unevenly distributed across colors. They were more commonly associated with Orange, Green, and Black, particularly in auditory or emotion-related conditions, suggesting that abstract conceptualization of color may rely more heavily on non-physical sensory cues.

Therefore, different colors evoke distinct adjective profiles, and that sensory modality plays a critical role in shaping the type of descriptors generated. While tactile stimulation primarily elicited appearance- and taste-related adjectives, auditory and emotional contexts were more effective in inducing emotional and abstract descriptors.

To further clarify how these aggregated patterns emerged across sensory modalities, the following subsections examine tactile, olfactory, auditory, and emotion-based descriptions separately.

#### 3.1.1 Tactile descriptions

It is found that most of the adjectives triggered by fruits are related to taste (Table B in Appendix C), such as “sour and sweet,” “fragrant,” and “crisp.” Moreover, even participant can also induce visual appearance words, such as “smooth,” “rough,” “hard,” “soft,” “fresh,” and “mature.” Because participant can touch the outline, texture, and surface material of fruits with their fingers, and feel their weight and size, they can easily imagine what they look like. Other categories include abstract (such as “dirty,” “dark,” and “light”) and emotional adjectives (such as “happy”).

#### 3.1.2 Olfactory descriptions

It is found that smell has the smallest number of adjectives (Table C in Appendix C), and that some words have been mentioned before. Most words focus on describing taste, such as “fragrant,” “smelly,” and “crisp,” while others evoke adjectives in the categories of emotion (“happy”), appearance (“mature” and “fresh”), and abstraction (“natural”).

#### 3.1.3 Auditory descriptions

It is found that sounds can easily induce emotional adjectives (Table D in Appendix C), such as “sad,” “happy,” and “scared” based on the fact that participants have no physical contact with objects. Most of the words produced have obviously different aspects and attributes from tactile and smell, such as taste (“bitter,” “juicy,” and “sour”), appearance (“mature”), and adjectives of abstract classes (“natural”).

#### 3.1.4 Emotional descriptions

Most of the adjectives are mainly abstract categories (Table E in Appendix C), such as “Spiritual,” “confident,” and “healthy.” The other categories are emotional adjectives. There are no taste, time, or appearance adjectives.

### 3.2 Recognition of color adjectives

Table 5 shows the representative adjectives for each color. The results reported below are based on one-sample *t*-tests and include only color-adjective pairs with mean ratings greater than 2 and statistical significance (*p* < 0.05; see Appendix D for full statistics). For red, the adjectives “emergency” [*t*_(14)_ = 2.78, *p* = 0.015] and “Happy” [*t*_(14)_ = 2.48, *p* = 0.027] were identified. For orange, significant adjectives included “sour and sweet” [*t*_(14)_ = 2.48, *p* = 0.027], “energetic” [*t*_(14)_ = 2.17, *p* = 0.048], “happy” [*t*_(14)_ = 2.48, *p* = 0.027], and “passionate” [*t*_(14)_ = 2.17, *p* = 0.048]. For yellow, the adjectives “bright” [*t*_(14)_ = 3.15, *p* = 0.007] and “beautiful” [*t*_(14)_ = 2.48, *p* = 0.027] met the criterion. For green, a broad range of adjectives showed significant results, including “outdoor” [*t*_(14)_ = 5.53, *p* = 0.001], “safe” [*t*_(14)_ = 3.57, *p* = 0.003], “free” [*t*_(14)_ = 4.18, *p* = 0.001], “peaceful” [*t*_(14)_ = 4.04, *p* = 0.001], “beautiful” [*t*_(14)_ = 9.54, *p* = 0.001], “fragrant” [*t*_(14)_ = 5.53, *p* = 0.001], “comfortable” [*t*_(14)_ = 9.54, *p* = 0.001], “happy” [*t*_(14)_ = 7.48, *p* = 0.001], and “fresh” [*t*_(14)_ = 4.18, *p* = 0.001]. For blue, significant adjectives included “happy” [*t*_(14)_ = 2.48, *p* = 0.027], “professional” [*t*_(14)_ [*t*_(14)_ = 3.15, *p* = 0.007], “soft” [*t*_(14)_ = 2.48, *p* = 0.027], “relaxed” [*t*_(14)_ = 2.81, *p* = 0.014], and “beautiful” [*t*_(14)_ = 2.81, *p* = 0.014]. For purple, only “rare” [*t*_(14)_ = 2.48, *p* = 0.027] showed a significant result. For white, the adjectives “Soft” [*t*_(14)_ = 2.48, *p* = 0.027], “dawn” [*t*_(14)_ = 2.48, *p* = 0.027], “cute” [*t*_(14)_ = 2.48, *p* = 0.027], “sweet” [*t*_(14)_ = 2.48, *p* = 0.027], and “plump” [*t*_(14)_ = 2.48, *p* = 0.027] were identified. For gray, however, no adjectives showed significant results (all *p* > 0.05). For black, the adjectives “dark” [*t*_(14)_ = 3.15, *p* = 0.007] and “low-profile” [*t*_(14)_ = 2.43, *p* = 0.029] met the criterion.

**Table 5:** Representative adjectives associated with each color.

Color	Adjective	Color	Adjective
Red	Emergency	Blue	Happy
	Happy		Professional
Orange	Sour and Sweet		Soft
	Energetic		Relaxed
	Happy		Beautiful
	Passionate	Purple	Rare
Yellow	Bright	White	Soft
	Beautiful		Dawn
Green	Outdoor		Cute
	Safe		Sweet
	Free		Plump
	Peaceful	Gray	-
	Beautiful		
	Fragrant		
	Comfortable		
	Happy	Black	Dark
	Fresh		Low-profile

**Figure 2:**
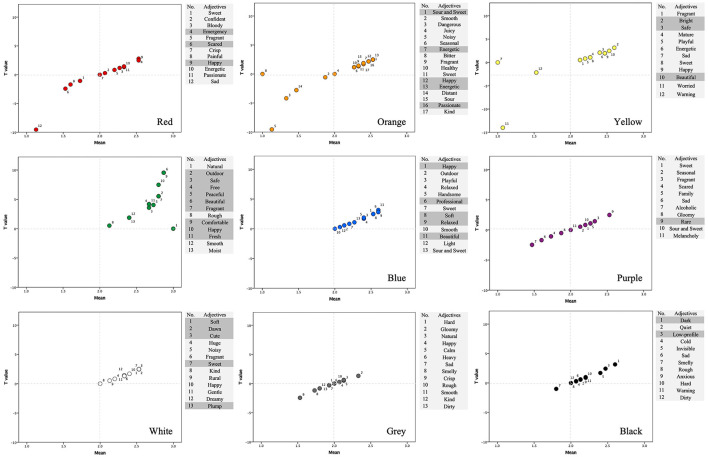
Distribution of color adjectives across mean ratings (*x*-axis) and T values (*y*-axis); the gray-shaded area denotes representative color adjectives.

Figure 2 visualizes the distribution of all color adjectives in a two-dimensional space, with mean ratings on the *x*-axis and corresponding T values on the *y*-axis. The gray-shaded region highlights representative color adjectives. Overall, the scatter plot shows a clear positive association between mean ratings and *t* values, indicating that adjectives with higher average ratings also tend to exhibit stronger statistical deviations from the test value. Adjectives exceeding the predefined criteria are thus visually distinguishable from the broader distribution, supporting the use of combined mean- and significance-based thresholds in identifying representative descriptors (Appendix D and Table 5 for corresponding statistical results).

## 4 Discussion

### 4.1 Advantages of multisensory stimulation induced color adjectives

The results of the present study indicate that multisensory stimulation facilitates the elicitation of diverse color-related descriptions among visually impaired participants, with distinct patterns of adjective use emerging across sensory contexts. Overall, participants more frequently produced appearance- and taste-related descriptors under tactile and olfactory conditions. In contrast, auditory and emotion-based contexts were more likely to elicit emotional and abstract descriptors. These findings suggest that non-visual sensory cues play an important role in shaping color-related descriptions, rather than relying on a single sensory modality (Barbiere et al., 2007; Lewis et al., 2013; Jonas et al., 2017; Marian et al., 2021; Ramsamy-Iranah et al., 2016; Reuterskiöld et al., 2011; Sun et al., 2018).

Further examination shows that the adjectives used by participants were largely grounded in sensory experience, including taste-related terms (e.g., “Sweet,” “Sour and sweet,” “Bitter”), appearance-related terms (e.g., “bright,” “dark,” “fresh,” “mature”), and tactile-related terms (e.g., “soft,” “hard,” “smooth”), with relatively fewer highly abstract or purely evaluative adjectives. This pattern is consistent with previous findings indicating that visually impaired individuals tend to focus on concrete and perceptible features when describing color-related experiences, rather than abstract aesthetic evaluations (Berman and Slobin, 1994; Kim et al., 2021). Such a descriptive tendency suggests that color concepts are commonly represented and organized through concrete sensory experiences.

Notably, when participants described the same color across different sensory modalities, the associated emotional descriptors varied and could even exhibit opposing emotional connotations. For example, red was described using both positive and negative emotional adjectives, such as “happy” and “dangerous.” This pattern indicates that color-related emotional descriptions are context-dependent and may reflect diverse interpretations shaped by the interaction of sensory cues and individual experience within cross-modal processing, rather than inherent emotional properties of the colors themselves (Amedi and Malach, 2014). In addition, emotional associations with color may be influenced by personal life experience and cultural background (Li et al., 2023). Taken together, these findings suggest that visually impaired participants are able to establish associations between colors and concrete descriptive terms within multisensory contexts, providing empirical insight into their color-related knowledge and perceptual representations.

In addition to tactile, auditory, and olfactory inputs, other types of bodily experiences may also help people understand color-related adjectives. Previous research suggests that language and meaning are closely linked to our overall sensory and bodily experiences (Barsalou, 2008; Glenberg, 2010). These include not only external senses, but also internal feelings such as body movement, physical comfort, and emotional states. This means that understanding color adjectives is not limited to specific sensory channels. Instead, it involves combining different types of experiences and organizing them through cognitive processes. In this study, multisensory stimuli provide useful cues, while cognition helps integrate these cues into meaningful language. This helps explain how visually impaired individuals can build an understanding of color without direct visual experience.

### 4.2 Types of color adjectives

Color adjectives of visually impaired people in this study can be divided into five categories: emotional, taste, appearance, time, and abstract adjectives. The reason for the classification of adjectives for visually impaired people is that visually impaired people cannot experience colors and visual elements through vision, so they must use other senses (such as tactile, olfactory, and auditory) to describe objects or environments, which can transform visual elements into language forms that can be understood by other senses, and these forms are usually expressed in the form of adjectives.

Emotional adjectives are influenced by multiple factors, including personal experience, cultural background, and environmental context. For individuals with visual impairments, emotional expressions are often grounded in concrete and sensory-based descriptions, which help facilitate understanding. Previous research suggests that visually impaired individuals tend to use more tangible and experience-based language to convey emotions, rather than relying solely on abstract emotional terms (Giraud et al., 2023; Gagliano, 2021).

Taste adjectives can be used to describe the taste characteristics of food, drinks, etc. For visually impaired people, smelling is a common way to perceive food. Therefore, descriptions of taste can help visually impaired people understand the taste characteristics of food using smell or taste adjectives (Ghatkamble et al., 2023; Riofrio-Grijalva et al., 2019). For example, to describe sweetness, you can use descriptions such as “smells sweet” or “tastes like sugar.”

Regarding time adjectives, visually impaired people can associate specific color adjectives with different seasons or times (Gegenfurtner et al., 2011). For example, “seasonal” and “dawn” can be used to describe a specific moment or event. These adjectives are meaningful to visually impaired people because they describe changes in time and weather, enabling them to better understand their surroundings. For example, when a visually impaired people hears “winter is coming” or “daybreak,” they can better understand what these words mean.

In addition, appearance adjectives are usually used to describe the appearance characteristics of objects, such as “fresh,” “smooth,” “mature,” “rough,” “dark,” “invisible,” and “dirty.” For visually impaired people, these adjectives are equivalent to descriptions of colors, which can help them establish images and concepts of objects, and further distinguish different objects (Edward, 2016). For example, when describing an apple, “fresh” enables visually impaired people to imagine the gloss and elasticity of the apple's surface, “smooth” allows visually impaired people to imagine the smoothness and softness of the apple's surface, and “mature” allows visually impaired people to imagine the sweetness of the apple flavor and the softness of the fruit.

Furthermore, visually impaired people's abstract descriptions of colors tend to express emotional aspects, such as “free,” “peaceful,” and “gloomy” (Gegenfurtner et al., 2011). Besides, visually impaired people are more likely to associate color adjectives with their life experiences, such as “professional,” “healthy,” and “safe” (Kim et al., 2021). These adjectives can help visually impaired people better understand the world around them and provide meaning and value in their lives.

### 4.3 Representative adjectives of color

Different colors evoked different emotional and semantic associations, as reflected in their representative adjectives. For visually impaired people, these associations are mainly learned through language and social experience, for example by hearing how others describe colors in daily conversation, education, and media, rather than through direct sensory experience (Kim et al., 2021; van Paridon et al., 2021). The following representative adjectives presented in this study are sample-specific and may not be generalizable to all visually impaired people.

Red was the only color that exhibited a bipolar emotional profile, eliciting both positive and negative representative adjectives such as “happy” and “emergency.” Notably, negative associations with red emerged exclusively under auditory stimulation, likely reflecting the influence of salient contextual cues such as emergency vehicle sounds, which are known to reinforce red's warning-related meanings (Elliot and Maier, 2014; Pravossoudovitch et al., 2014).

Orange was primarily associated with taste-related descriptors and high-arousal positive emotions, including “sour and sweet,” “energetic,” and “happy.” This pattern is consistent with previous findings linking orange to food-related experiences and energetic affect through stable crossmodal correspondences (Barbiere et al., 2007; Spence, 2011; Sun et al., 2018).

Yellow yielded representative adjectives related to brightness and positive evaluation, such as “bright” and “beautiful.” These associations reflect yellow's strong linkage with high luminance and perceptual salience, and appear to be maintained through language and cultural experience even in the absence of direct visual perception (Gegenfurtner and Rieger, 2000; Küller et al., 2006).

Green produced the most semantically coherent set of representative adjectives, encompassing nature-related concepts (e.g., “fresh,” “outdoor”), positive affect (e.g., “happy,” “peaceful”), and comfort- and safety-related meanings (e.g., “comfortable,” “safe”). This pattern aligns with prior research showing that green is consistently associated with nature, relaxation, and low-arousal positive emotions across sensory modalities (Küller et al., 2006; Spence, 2011).

Blue was associated with low-arousal positive affect and evaluative meanings such as “relaxed,” “soft,” and “professional,” supporting its well-documented links to calmness, stability, and trustworthiness (Barbiere et al., 2007; Küller et al., 2006). These associations may be further reinforced by crossmodal correspondences with low-intensity sensory attributes (Sun et al., 2018).

Purple elicited only one representative adjective, “rare,” suggesting limited and highly abstract semantic associations. This finding is consistent with previous research indicating that purple-related meanings are primarily acquired through language and cultural conventions rather than frequent sensory experience (Josserand et al., 2021).

White was associated with gentle and concrete descriptors related to texture and affect, including “soft” and “sweet,” reflecting linguistic and cultural associations with softness and positive affect (Na and Suk, 2014). In contrast, no representative adjectives were identified for gray, possibly due to the wide dispersion and semantic inconsistency of appearance-related descriptors elicited under tactile conditions. Finally, black was characterized by abstract evaluative meanings such as “dark” and “low-profile,” reflecting associations with low luminance and socially learned color semantics rather than direct sensory attributes (Gegenfurtner and Rieger, 2000; Saysani et al., 2018).

## 5 Conclusions

This study explores visually impaired people's knowledge and understanding of color, as well as changes in their color perception. The research results show that visually impaired people's understanding of color can indeed be related to color adjectives produced by other sensory stimulations such as tactile and auditory. The adjectives used by visually impaired people can be mapped to the correct colors, and they are even more sensitive to auditory and tactile stimulation than is the case for sighted people. However, due to the lack of visual stimulation, visually impaired people's understanding of colors may be affected by factors such as language and culture, and their induced vocabulary size and word-making ability are weaker than those of sighted people. The color preferences and emotional reactions of visually impaired people may be affected by factors such as personal experience and cultural background.

These findings provide profound contributions to our understanding of color and perception in visually impaired people. First of all, in terms of exploring the color preferences of visually impaired people, research has pointed out that the preference for black may be related to the degree of visual impairment. The reasons for this preference can be attributed to aspects such as high contrast, simplicity, and a sense of stability. This research provides a practical reference for designing visually impaired-friendly environments and products, emphasizes understanding the needs of visually impaired people, and helps improve their quality of life. Secondly, in terms of the advantages of multisensory stimulation induced color adjectives, research has broken through the limitations of past understanding of color and revealed the diverse understanding of color by visually impaired people through media contact with sensory stimulation such as tactile and hearing, thus providing inspiration for the field of education, special needs education, and artistic creation, while also emphasizing the importance of designing sensory aids. In terms of the types of color adjectives for the visually impaired people, this study subdivided them into emotion, taste, appearance, time, and abstract adjectives. This classification provides us with a more detailed understanding and enables us to better meet the language expression needs of the visually impaired people. At the same time, it also highlights the greater reliance that visually impaired people have on specific sensory stimulation when perceiving the world, which is crucial for developing more effective ways of communicating. Finally, with regard to auditory-evoked emotional adjectives, research has highlighted the sensitivity and richness of auditory stimuli in visually impaired people. This insight not only broadens our understanding of perceptual experience, but also provides suggestions that can help design emotionally rich environments and experiences, with substantial significance for improving the emotional participation and life satisfaction of visually impaired people. Overall, these findings expand our understanding of the perceptions and expressions of visually impaired people, provide us with a deeper perspective, and hopefully lead to more design and educational practices that are closer to the needs and experiences of visually impaired people. Despite these contributions, this study has several limitations. First, the sample size of the interview study was relatively small (*n* = 6), which may limit the generalizability of the findings. In addition, the two experiments involved people with different levels of visual experience, and therefore the results should be interpreted as exploratory and complementary rather than directly comparable. Future studies with larger and more homogeneous samples are needed to further validate and extend the present findings.

## Data Availability

The original contributions presented in the study are included in the article/Supplementary material, further inquiries can be directed to the corresponding author.
