# Supplementary material for: Adjectives improve color perception in visually impaired people through multisensory stimulation
Source: Front Psychol. 2026 May 18;17:1718682. doi: 10.3389/fpsyg.2026.1718682 (PMC13223054; doi:10.3389/fpsyg.2026.1718682)
Supplement: Supplementary file 1 [file Data_Sheet_1.docx]

**Appendix A.** Participant Information

Table A. Participants in Experiment 1

| NO. | Gender | Age | Classification of Visual Impairment* | Onset | Cause |
| --- | --- | --- | --- | --- | --- |
| 1 | Male | 14 | Level 4 (Blindness) | Congenital | Congenital retinal disorder |
| 2 | Male | 13 | Level 4 (Blindness) | Congenital | Retinopathy of prematurity (right eye lens replaced; left eye corneal transplant; light perception retained) |
| 3 | Female | 16 | Level 4 (Blindness) | Congenital | Congenital optic nerve hypoplasia |
| 4 | Female | 17 | Level 5 (Total blindness) | Congenital | Retinopathy of prematurity |
| 5 | Female | 13 | Level 5 (Total blindness) | Congenital | Retinitis pigmentosa (minimal light perception) |
| 6 | Female | 12 | Level 5 (Total blindness) | Congenital | Elevated intraocular pressure caused by corneoscleralization |

*Note*: *Visual impairment levels follow WHO classification.

Table B. Participants in Experiment 2

| NO. | Gender | Age | Classification of Visual Impairment | Onset | ID |
| --- | --- | --- | --- | --- | --- |
| 1 | Female | 17 | Level 5 (Total blindness) | Congenital | Retinopathy of prematurity |
| 2 | Female | 13 | Level 5 (Total blindness) | Congenital | Retinitis pigmentosa |
| 3 | Male | 14 | Level 4 (Blindness) | Congenital | Congenital retinal disorder |
| 4 | Female | 16 | Level 4 (Blindness) | Congenital | Congenital optic nerve hypoplasia |
| 5 | Male | 13 | Level 4 (Blindness) | Congenital | Retinopathy of prematurity |
| 6 | Female | 11 | Level 5 (Total blindness) | Congenital | Optic nerve and retinal hypoplasia; aniridia |
| 7 | Male | 19 | Level 5 (Total blindness) | Congenital | Congenital retinal hypoplasia; aniridia |
| 8 | Male | 12 | Level 2 (Moderate visual impairment) | Congenital | Congenital optic nerve hypoplasia |
| 9 | Male | 9 | Level 2 (Moderate visual impairment) | Congenital | Shaken baby syndrome |
| 10 | Female | 8 | Level 5 (Total blindness) | Congenital | Retinopathy of prematurity |
| 11 | Female | 17 | Level 4 (Blindness) | Congenital | Retinopathy of prematurity |
| 12 | Female | 17 | Level 4 (Blindness) | Congenital | Cortical visual impairment due to prematurity |
| 13 | Male | 20 | Level 5 (Total blindness) | Congenital | Retinal detachment due to prematurity |
| 14 | Female | 17 | Level 2 (Moderate visual impairment) | Congenital | Congenital optic nerve defect with progressive degeneration |
| 15 | Male | 18 | Level 2 (Moderate visual impairment) | Congenital | Premature birth with hypoxia |

*Note*: *Visual impairment levels follow WHO classification.
