# Supplementary material for: Adjectives improve color perception in visually impaired people through multisensory stimulation
Source: Front Psychol. 2026 May 18;17:1718682. doi: 10.3389/fpsyg.2026.1718682 (PMC13223054; doi:10.3389/fpsyg.2026.1718682)
Supplement: Supplementary file 2 [file Data_Sheet_2.docx]

*Appendix B.* Interview outline of visually impaired children’s color concepts and experiences

| Part 1: Description of basic color concepts | | |
| --- | --- | --- |
| 1. What is your favorite color and why do you like it? 2. How to know color? How do your friends, family, and teachers describe colors. 3. Do you usually talk about colors? Under what circumstances? 4. How does school teach you to understand colors? | | |
| Part 2: Color sensory description | | Color and Prop |
| 1. Describe colors through tactile | Have the visually impaired child hold the prop and describe its feel and color. For example, let him hold an apple, guess what it is, and ask him to describe his feelings and tell similar experiences in the past. | Red: Apple  Orange: Orange  Yellow: Banana  Green: Leaves  Blue: Blueberry  Purple: Grape  White: Marshmallow  Gray: Stone  Black: Charcoal |
| 1. Describe colors through olfactory | For example, give a visually impaired child an orange, tell him that oranges are orange, ask them to pay attention to the smell and taste, and ask them to describe their feelings and tell similar experiences in the past. |  |
| 1. Describe colors through auditory | For example, tell a visually impaired child that he should think of red when hearing a siren, because red is usually used to attract people's attention, and ask him to describe his feelings and describe similar experiences in the past. | Red: Fire truck  Orange: Horn  Yellow: Duck  Green: Rustling grassland  Blue: Sea waves  Purple: Egyptian music  White: Rooster crowing  Gray: Elephant  Black: Nightingale |
| Part 3: Color emotional description | |  |
| Describe the emotions and feelings the color brings to you | Introduce the most familiar color feelings to visually impaired children, such as red, which is often associated with anger, excitement, vitality, etc., and let them describe the feelings and tell similar experiences in the past. | |
