# Supplementary material for: Adjectives improve color perception in visually impaired people through multisensory stimulation
Source: Front Psychol. 2026 May 18;17:1718682. doi: 10.3389/fpsyg.2026.1718682 (PMC13223054; doi:10.3389/fpsyg.2026.1718682)
Supplement: Supplementary file 3 [file Data_Sheet_3.docx]

*Appendix C*. Interview results on the color description through tactile, olfactory, hearing, and emotion (the numbers in parentheses are frequencies).

Table A. The overall interview coding results

| Color | Adjective |
| --- | --- |
| Red | Sweet(6)/Happy(4)/Energetic(4)/Emergency(3)/Fragrant(3)/Sad(2)/  Confident(2)/Passionate(2)/Crisp(2)/Bloody(1)/Fresh(1)/Painful(1) |
| Orange | Energetic(6)/Healthy(5)/Happy(4)/Sour and Sweet(4)/Passionate(3)/ Kind(3)/Distant(3)/Sweet(2)/Dangerous(2)/Juicy(2)/Seasonal(1)/  Smooth(1)/Sour(1)/Bitter(1)/Noisy(1)/Fragrant(1) |
| Yellow | Warning(8)/Sweet(4)/Mature(4)/Fragrant(1)/Playful(1)/Happy(1)/  Worried(1)/Beautiful(1)/Energetic(1)/Bright(1)/Safe(1)/Scared(1) |
| Green | Fresh(5)/Natural(5)/Safe(4)/Free(3)/Beautiful(3)/Rough(2)/Fragrant(1)/  Moist(1)/Smooth(1)/Outdoor(1)/Happy(1)/Fresh(1)/Comfortable(1)/  Peaceful(1) |
| Blue | Sweet(3)/Happy(3)/Playful(2)/Smooth(2)/Professional(2)/Outdoor(2)/  Relaxed(2)/Soft(1)/Light(1)/Sour and Sweet(1)/Beautiful(1)/  Handsome(1)/Natural(1) |
| Purple | Sweet(4)/Fragrant(3)/Scared(2)/Seasonal(1)/Alcoholic(1)/Sour and Sweet(1)/Gloomy(1)/Family(1)/Rare(1)/Melancholy(1) |
| White | Soft(5)/Sweet(4)/Dreamy(4)/Kind(3)/Fragrant(2)/Happy(2)/Rural(2)/  Dawn(2)/Noisy(2)/Gentle(1)/Cute(1)/Plump(1) |
| Grey | Smelly(4)/Smooth(3)/Hard(3)/Heavy(3)/Calm(2)/Rough(1)/Crisp(1)/  Natural(1)/Dirty(1)/Huge(1)/Kind(1)/Happy(1)/Gloomy(1)/Scared(1) |
| Black | Smelly(3)/Cold(3)/Low-Profile(2)/Dirty(2)/Dark(2)/Rough(2)/Hard(1)/  Invisible(1)/Quiet(1)/Sad(1)/Anxious(1)/Scared/(1)Anxious(1) |

Table B. Interview coding results in color description of tactile

| Color | Adjective | Extract important content parts | |
| --- | --- | --- | --- |
| Red | Happy(2)/Sweet(2)/Crisp(1) | | I feel in a good mood (S1)/ It tastes sweet ...I feel happy (S2) /It tastes crunchy (S3)/It tastes sweet (S4) |
| Orange | Sour and Sweet(4)/Juicy(1)/  Seasonal(1)/Happy(1)/  Sweet(1)/Smooth(1)/Sour(1) | | ...It tastes sweet and sour when you eat it (S1)/ Sour and sweet ...soft and squirting juice ...Oranges are only available in winter (S2)/It tastes sweet and slightly sour ...you will be very happy (S3)/ sweet ... smooth to the touch (S4)/The oranges in season are very sweet, but the ending is very sour (S5)/It tastes sour (S6) |
| Yellow | Mature(2)/Sweet(2)/  Fragrant(1) | | Very fragrant and ready to eat when ripe ...it tastes sweet (S2)/it tastes sweet (S3)/the green color is immature (S5)/ it tastes sweet (S6) |
| Green | Fresh(3)/Rough(2) | | It can be felt that it is not withered, so it is judged that it is green and has a thick feeling (S2)/It feels thick(S3)/fresh leaves...have roots (S4)/…I have touched dead fallen leaves, So it can be distinguished as green (S5) |
| Blue | Smooth(2)/Soft(1)/Light(1) | | The skin feels smooth (S1)/small and slippery (S2)/ soft and easy to crush (S4)/ light(S5) |
| Purple | Sour and Sweet(1)/  Seasonal(1)/Sweet(1) | | sweet and sour feeling (S1)/Purple color lets him know what season is coming (S2)/ so sweet ... (S3) |
| White | Soft(5)/Sweet(1)/Plump(1) | | sweet, soft ... very soft…(S1)/ soft (S2)/ soft to touch (S3.S4)/ surging marshmallow(S5)/ soft to touch (S6) |
| Grey | Smooth(3)/Hard(3)/Heavy(3)/Rough(1)/Crisp(1) | | It feels hard, heavy when picked up, and crisp when knocked (S1)/It feels thick...it feels heavy and hard when picked up (S2)/It feels hard and heavy when picked up (S3)/It feels smooth ( S4.S5.S6 ) |
| Black | Rough(2)/Dirty(1)/Dark(1)/  Hard(1) | | Scratchy, dirty hands...dark, black (S1)/ pinky to the touch (S2)/ hard to the touch (S3)/ thick to the touch (S4)/ rusty (S6) |

Note: The number S is the participant’s code, and the number is the order of the participants. For example, S1 is the first participant.

Table C. Interview coding results in color description of olfactory

| Color | Adjective | Extract important content parts |
| --- | --- | --- |
| Red | Fragrant(3)/Sweet(4)/Crisp(1)/Fresh(1)/Happy(1) | fragrant, it tastes sweet and has a crunchy feeling (S1)/ sweet feeling (S2)/smells sweet (S3)/ fragrant, it smells like fresh apples, it will feeling very happy (S4)/ smells and sweets (S6) |
| Orange | Bitter(1)/Juicy(1)/Sweet(1)/  Energetic(1)/Fragrant(1) | sometimes it tastes bitter and dripping with juice (S1)/sweet taste (S2)/very energetic(S3)/spicy(S4) |
| Yellow | Warning(4)/Mature(2)/  Sweet(2) | very warning... it's soft and ripe(S1)/ very warning, if the sourness means the banana is not ripe (S2)/ very warning taste (S3)/ sweet(S4)/a little bit sweet (S5)/ sweet and warning (S6) |
| Green | Fresh(2)/Natural(2)/  Fragrant(1) | no dead leaves (S2)/ the smell of mango trees (S3)/ the smell of grass (S4)/a slightly fragrant smell (S5)/ fresh leaves (S6) |
| Blue | Sweet(3)/Sour and Sweet(1) | it tastes very sweet (S1)/ it tastes sour and sweet(S2)/ it tastes sweet(S4)/ |
| Purple | Sweet(4)/Fragrant(3)/  Alcoholic(1) | sweet (S1.S6)/ sweet taste (S2)/smells very fragrant (S3)/ sweet(S4)/ sweet taste, a bit like wine(S5) |
| White | Sweet(3)/Fragrant(2)/  Happy(1) | fragrant and sweet, i don’t feel anything about white (S1)/ sweet smell, very happy (S2)/ very fragrant (S3.S6)/ sweet (S4.S5) |
| Grey | Smelly(4)/Natural(1)/Dirty(1) | it smells like dirt ...the hands feel dirty and unclean (S1)/ smells like grass (S3)/smells stinky (S4)/smells like dirt (S6) |
| Black | Smelly(3)/Dark(1)/  Invisible(1) | dark, invisible (S1)/smells stinky (S4.S5.S6) |

Table D. Interview coding results in color description of auditory

| Color | Adjective | Extract important content parts |
| --- | --- | --- |
| Red | Emergency(3)/Sad(2)/  Scared(1) | that something happened, I'm very sad, I need to put out a fire (S1)/I feel scared (S2)/I need something to put out a fire (S4)/ I'll hear it outside the hospital (S5) |
| Orange | Distant(3)/Dangerous(2)/  Noisy(1) | it's very similar to someone who is about to quarrel or fight (S1)/the sound feels dangerous (S2)/hears this sound and needs to stay away (S3)/hears this sound and needs to let the car pass (S4)/knows to avoid, knows it is dangerous sound(S6) |
| Yellow | Playful(1)/Happy(1) | the feeling of playing, the feeling of performing, you will feel very happy (S1) |
| Green | Moist(1)/Smooth(1)/  Natural(2)/Outdoor(1) | the grassland feels wet to the touch and slippery to walk on (S1)/ the chirping of cicadas in nature (S2)/I think they are maple leaves (S5)/ I heard it in the park (S6) |
| Blue | Happy(2)/Playful(2)/  Outdoor(2)/Relaxed(1) | I have touched the sea water and the waves will come up... I feel very good (S1)/ it is fun and I feel like I can go out (S2)/ I feel very good when I go out to play(S4)/I have been to the coast and it is fun...I am very playful, very relaxing, and very happy( S5)/ it’s fun, you can wash your feet (S6) |
| Purple | Gloomy(1)/Scared(1) | gloomy feeling... very scared (S5) |
| White | Rural(2)/Dawn(2)/Noisy(2) | in the countryside (S1)/ I feel like it’s dawn and I can get up (S2) / I hear it in the morning, it’s very noisy(S4)/it’s very noisy in the morning, paired with dawn(S5)/ you can hear it at noon, it’s very noisy(S6) |
| Grey | Huge(1) | the long nose feels very big (S1) |
| Black | Quiet(1)/Sad(1) | didn't hear any sound at night (S1)/ terrifying sound (S5) |

Table E. Interview coding results in color description of emotion

| Color | Adjective | Extract important content parts |
| --- | --- | --- |
| Red | Energetic(4)/Confident(2)/  Passionate(2)/Happy(1)/ Bloody(1)/Painful(1) | full of energy ...feeling confident (S1)/very happy, passionate, energetic, confident (S2)/very passionate (S3)/ full of energy (S4)/red blood, painful(S5) |
| Orange | Energetic(5)/Healthy(5)/  Passionate(3)/Happy(3)/  Kind(3) | energetic, kind, and healthy (S1)/very energetic, cheerful, kind, and friendly (S2)/ energetic, healthy, and kind(S3)/ energetic, happy (S4)/very energetic, healthy(S6) |
| Yellow | Warning(4)/Worried(1)/  Beautiful(1)/Energetic(1)/  Bright(1)/Safe(1)/Scared(1) | warning effect (S1.S3)/ feeling of worry (S2)/ beautiful, warning feeling (S4)/very energetic... bright future. it feels like a warning, and the guide volunteers are also yellow (S5)/it’s scared, and it feels like a warning (S6) |
| Green | Safe(4)/Free(3)/Beautiful(3)/Happy(1)/Fresh(1)/Natural(1)  /Comfortable(1)Peaceful(1)/ | happy, free, fresh, comfortable, safe (S2)/ security, peaceful feeling (S3)/ beautiful feeling, safe, free, natural (S4)/very beautiful, some flowers are green (S5) / beautiful, safe, free (S6) |
| Blue | Beautiful(1)/Professional(2)/  Happy(1)/Handsome(1)/  Natural(1)/Relaxed(1) | very happy ... very beautiful (S2)/ professional feeling (S3)/ handsome and professional (S4)/ relaxing and leisurely feeling, thinking of the seaside (S5) |
| Purple | Family(1)/Rare(1)/Scared(1)/  Melancholy(1)/ | of going home ... there are few purple things (S2)/ melancholy feeling (S3)/ elegant (S4)/more scared(S6) |
| White | Dreamy(4)/Kind(3)/Happy(1)/Gentle(1)/Cute(1) | dreamy (S1)/ very happy, happy, and gentle (S2)/ dreamy, kind (S3)/ cute, dreamy, kind (S4)/ dreamy, kind (S6) |
| Grey | Calm(2)/Kind(1)/Happy(1)/  Gloomy(1)/Scared(1) | calm(S1.S2)/ kind (S3)/very happy, the dog is gray (S4)/ gloomy(S5)/scared |
| Black | Cold(3)/Dark(2)/Low-Profile (2)/Anxious(2)/ Scared/(1) | dark, low-profile, very cold (S1)/ very annoying, a little cold (S2)/ dark, like hell (S5)/very scared, anxious, cold (S6) |
