# Supplementary material for: Adjectives improve color perception in visually impaired people through multisensory stimulation
Source: Front Psychol. 2026 May 18;17:1718682. doi: 10.3389/fpsyg.2026.1718682 (PMC13223054; doi:10.3389/fpsyg.2026.1718682)
Supplement: Supplementary file 4 [file Data_Sheet_4.docx]

*Appendix* *D.* The result of recognition of color adjectives for visually impaired children using one-sample t-test. The grey-shaded region highlights representative color adjectives.

| Color | Adjective | Mean | SD | T value | Sig. |
| --- | --- | --- | --- | --- | --- |
| Red | Sweet | 1.73 | 0.96 | -1.08 | 0.301 |
|  | Confident | 2.07 | 0.96 | 0.27 | 0.792 |
|  | Bloody | 2.27 | 0.88 | 1.17 | 0.262 |
|  | Emergency | 2.53 | 0.74 | 2.78 | 0.015 * |
|  | Fragrant | 2.20 | 0.94 | 0.82 | 0.424 |
|  | Scared | 1.53 | 0.74 | -2.43 | 0.029 * |
|  | Crisp | 2.00 | 1.00 | 0.00 | 1.000 |
|  | Painful | 1.60 | 0.91 | -1.70 | 0.111 |
|  | Happy | 2.53 | 0.83 | 2.48 | 0.027 * |
|  | Energetic | 2.33 | 0.98 | 1.32 | 0.207 |
|  | Passionate | 2.33 | 0.90 | 1.44 | 0.173 |
|  | Sad | 1.13 | 0.35 | -9.54 | 0.000 ** |
| Orange | Sour and Sweet | 2.53 | 0.83 | 2.48 | 0.027 * |
|  | Smooth | 1.87 | 0.92 | -0.56 | 0.582 |
|  | Dangerous | 1.33 | 0.62 | -4.18 | 0.001 ** |
|  | Juicy | 2.00 | 1.00 | 0.00 | 1.000 |
|  | Noisy | 1.13 | 0.35 | -9.54 | 0.000 ** |
|  | Seasonal | 2.27 | 0.88 | 1.17 | 0.262 |
|  | Energetic | 2.47 | 0.83 | 2.17 | 0.048 * |
|  | Bitter | 1.00 | 0.00^a^ | - | - |
|  | Fragrant | 2.27 | 0.88 | 1.17 | 0.262 |
|  | Healthy | 2.33 | 0.98 | 1.32 | 0.207 |
|  | Sweet | 2.33 | 0.90 | 1.44 | 0.173 |
|  | Happy | 2.53 | 0.83 | 2.48 | 0.027 * |
|  | Energetic | 2.53 | 0.83 | 2.48 | 0.027 * |
|  | Distant | 1.47 | 0.74 | -2.78 | 0.015 * |
|  | Sour | 2.40 | 0.91 | 1.70 | 0.111 |
|  | Passionate | 2.47 | 0.83 | 2.17 | 0.048 * |
|  | Kind | 2.40 | 0.83 | 1.87 | 0.082 |
| Yellow | Fragrant | 2.13 | 0.99 | 0.52 | 0.610 |
|  | Bright | 2.60 | 0.74 | 3.15 | 0.007 ** |
|  | Safe | 2.40 | 0.74 | 2.10 | 0.054 * |
|  | Mature | 2.27 | 0.96 | 1.08 | 0.301 |
|  | Playful | 2.20 | 0.94 | 0.82 | 0.424 |
|  | Energetic | 2.47 | 0.92 | 1.97 | 0.068 |
|  | Sad | 1.00 | 0.00^a^ | - | - |
|  | Sweet | 2.27 | 0.96 | 1.08 | 0.301 |
|  | Happy | 2.47 | 0.92 | 1.97 | 0.068 |
|  | Beautiful | 2.53 | 0.83 | 2.48 | 0.027 * |
|  | Worried | 1.07 | 0.26 | -14.00 | 0.000 ** |
|  | Warning | 1.53 | 0.83 | -2.17 | 0.048 * |
| Green | Natural | 3.00 | 0.00^a^ | - | - |
|  | Outdoor | 2.80 | 0.56 | 5.53 | 0.000 ** |
|  | Safe | 2.67 | 0.72 | 3.57 | 0.003 ** |
|  | Free | 2.67 | 0.62 | 4.18 | 0.001 ** |
|  | Peaceful | 2.73 | 0.70 | 4.04 | 0.001 ** |
|  | Beautiful | 2.87 | 0.35 | 9.54 | 0.000 ** |
|  | Fragrant | 2.80 | 0.56 | 5.53 | 0.000 ** |
|  | Rough | 2.13 | 0.99 | 0.52 | 0.610 |
|  | Comfortable | 2.87 | 0.35 | 9.54 | 0.000 ** |
|  | Happy | 2.80 | 0.41 | 7.48 | 0.000 ** |
|  | Fresh | 2.67 | 0.62 | 4.18 | 0.001 ** |
|  | Smooth | 2.40 | 0.83 | 1.87 | 0.082 |
|  | Moist | 2.40 | 0.83 | 1.87 | 0.082 |
| Blue | Happy | 2.53 | 0.83 | 2.48 | 0.027 * |
|  | Outdoor | 2.13 | 0.92 | 0.56 | 0.582 |
|  | Playful | 2.40 | 0.91 | 1.70 | 0.111 |
|  | Relaxed | 2.40 | 0.91 | 1.70 | 0.111 |
|  | Handsome | 2.40 | 0.83 | 1.87 | 0.082 |
|  | Professional | 2.60 | 0.74 | 3.15 | 0.007 ** |
|  | Sweet | 2.20 | 0.94 | 0.82 | 0.424 |
|  | Soft | 2.53 | 0.83 | 2.48 | 0.027 * |
|  | Relaxed | 2.60 | 0.83 | 2.81 | 0.014 * |
|  | Smooth | 2.00 | 1.00 | 0.00 | 1.000 |
|  | Beautiful | 2.60 | 0.83 | 2.81 | 0.014 * |
|  | Light | 2.07 | 0.96 | 0.27 | 0.792 |
|  | Sour and Sweet | 2.27 | 0.96 | 1.08 | 0.301 |
| Purple | Sweet | 2.20 | 0.94 | 0.82 | 0.424 |
|  | Seasonal | 2.13 | 0.99 | 0.52 | 0.610 |
|  | Fragrant | 2.33 | 0.90 | 1.44 | 0.173 |
|  | Scared | 1.73 | 0.96 | -1.08 | 0.301 |
|  | Family | 2.27 | 0.96 | 1.08 | 0.301 |
|  | Sad | 1.60 | 0.91 | -1.70 | 0.111 |
|  | Alcoholic | 1.47 | 0.83 | -2.48 | 0.027 * |
|  | Gloomy | 1.87 | 0.99 | -0.52 | 0.610 |
|  | Rare | 2.53 | 0.83 | 2.48 | 0.027 * |
|  | Sour and Sweet | 2.27 | 0.96 | 1.08 | 0.301 |
|  | Melancholy | 2.00 | 1.00 | 0.00 | 1.000 |
| White | Soft | 2.53 | 0.83 | 2.48 | 0.027 * |
|  | Dawn | 2.53 | 0.83 | 2.48 | 0.027 * |
|  | Cute | 2.53 | 0.83 | 2.48 | 0.027 * |
|  | Huge | 2.20 | 0.94 | 0.82 | 0.424 |
|  | Noisy | 1.07 | 0.26 | -14.00 | 0.000 ** |
|  | Fragrant | 2.33 | 0.90 | 1.44 | 0.173 |
|  | Sweet | 2.53 | 0.83 | 2.48 | 0.027 * |
|  | Kind | 2.00 | 1.00 | 0.00 | 1.000 |
|  | Rural | 2.13 | 0.99 | 0.52 | 0.610 |
|  | Happy | 2.40 | 0.91 | 1.70 | 0.111 |
|  | Gentle | 2.33 | 0.98 | 1.32 | 0.207 |
|  | Dreamy | 2.33 | 0.98 | 1.32 | 0.207 |
|  | Plump | 2.53 | 0.83 | 2.48 | 0.027 * |
| Grey | Hard | 2.00 | 1.00 | 0.00 | 1.000 |
|  | Gloomy | 2.33 | 0.98 | 1.32 | 0.207 |
|  | Natural | 2.13 | 0.99 | 0.52 | 0.610 |
|  | Happy | 2.07 | 0.96 | 0.27 | 0.792 |
|  | Calm | 2.13 | 0.92 | 0.56 | 0.582 |
|  | Heavy | 2.00 | 0.93 | 0.00 | 1.000 |
|  | Sad | 1.93 | 0.96 | -0.27 | 0.792 |
|  | Smelly | 1.73 | 0.88 | -1.17 | 0.262 |
|  | Crisp | 1.53 | 0.74 | -2.43 | 0.029 * |
|  | Rough | 2.13 | 0.83 | 0.62 | 0.546 |
|  | Smooth | 2.13 | 0.83 | 0.62 | 0.546 |
|  | Kind | 1.80 | 0.94 | -0.82 | 0.424 |
|  | Dirty | 1.93 | 0.88 | -0.29 | 0.774 |
| Black | Dark | 2.60 | 0.74 | 3.15 | 0.007 ** |
|  | Quiet | 2.13 | 0.92 | 0.56 | 0.582 |
|  | Low-profile | 2.47 | 0.74 | 2.43 | 0.029 * |
|  | Cold | 2.07 | 0.80 | 0.32 | 0.751 |
|  | Invisible | 2.40 | 0.91 | 1.70 | 0.111 |
|  | Sad | 2.13 | 0.83 | 0.62 | 0.546 |
|  | Smelly | 1.80 | 0.78 | -1.00 | 0.334 |
|  | Rough | 2.00 | 0.85 | 0.00 | 1.000 |
|  | Anxious | 2.20 | 0.86 | 0.90 | 0.384 |
|  | Hard | 2.20 | 0.86 | 0.90 | 0.384 |
|  | Warning | 2.20 | 0.78 | 1.00 | 0.334 |
|  | Dirty | 2.07 | 0.96 | 0.27 | 0.792 |

*Note:* *p<0.05; **p<0.01

^a^ T value cannot be computed because the standard deviation is 0.
